# Medical Management of Tenosynovial Giant Cell Tumor

**DOI:** 10.1007/s11912-025-01679-x

**Published:** 2025-05-20

**Authors:** Emanuela Palmerini, Jonathan C. Trent, Francis John Hornicek Jr

**Affiliations:** 1 Sylvester Comprehensive Cancer Center, University of Miami, 1475 NW 12th Ave, Miami, FL 33136 USA; 2 Miller School of Medicine, University of Miami, Miami, FL USA

**Keywords:** Colony-stimulating factor 1 receptor, Emactuzumab, Pexidartinib, Pimicotinib, Tenosynovial giant cell tumor, Vimseltinib

## Abstract

**Purpose of Review:**

Diffuse tenosynovial giant cell tumor (D-TGCT) is a benign neoplasm with locally aggressive potential of the synovium, bursae, and tendon sheaths. This review summarizes the current treatment landscape for D-TGCT, with a focus on systemic therapies.

**Recent Findings:**

Surgery is the primary treatment option for tenosynovial giant cell tumor (TGCT), but there is a high risk of recurrence and associated morbidity, particularly for patients with advanced D-TGCT. Systemic therapies targeting the colony-stimulating factor 1 receptor (CSF1R) have resulted in positive tumor response, improved function, and decreased symptoms. For an alternative to surgery, the CSF1R inhibitors pexidartinib and vimseltinib are approved in the United States for TGCT, and other CSF1R inhibitors are in clinical development.

**Summary:**

CSF1R inhibitors represent a significant evolution in therapeutic strategies for D-TGCT. The potential risks and benefits of available treatments should be carefully considered in collaboration with a bone tumor–experienced, multidisciplinary team to determine the best course of care. Increased D-TGCT awareness and support through patient advocacy groups have helped to reshape the patient journey.

## Introduction

Tenosynovial giant cell tumor (TGCT), formerly pigmented villonodular synovitis or giant cell tumor of tendon sheath, is a rare, benign neoplasm developing from the synovial tissue of joints, bursae, and tendon sheaths [1, 2]. TGCT is classified into 2 types based on growth pattern and location. Localized TGCT (L-TGCT) typically presents as a single lesion or nodule in the knee and hip, as well as in smaller joints, often in the hands and wrists [1]. Diffuse TGCT (D-TGCT) presents as multiple nodules mainly in larger joints, such as the knee, ankle, and hip, with aggressive growth and extensive involvement of the synovium [1, 2]. Disease onset usually occurs in younger adults (aged 35–50 years) [1]. Data on the incidence rates of TGCT are limited, ranging from approximately 10–50 per million for L-TGCT and approximately 5–10 per million for D-TGCT [1, 3, 4].

The pathophysiology of TGCT is characterized by genetic rearrangements in colony-stimulating factor 1 (*CSF1*) [5, 6]. CSF1 regulates macrophage differentiation, proliferation, and survival [5, 7]. Different chromosomal rearrangements have been reported in TGCT, such as a t(1;2) translocation linking *CSF1* to the *COL6A3* collagen promoter, which results in deletion of the 3’ untranslated region and overexpression of CSF1 [5, 6, 8]. CSF1 upregulation stimulates the proliferation of neoplastic cells within the tumor and the recruitment of non-neoplastic macrophages that express the CSF1 receptor (CSF1R) to the synovium [5]. CSF1 binding to CSF1R activates kinase signaling pathways that drive tumor growth. New and emerging pharmacologic therapies for TGCT have focused on targeting the CSF1/CSF1R signaling pathway.

TGCT is a complex disease with a broad spectrum of severity, ranging from mild symptoms to debilitating joint pain, stiffness, swelling, and reduced range of motion that can impact quality of life (QoL) [9–11]. A study of the TGCT Observational Platform Project (TOPP) registry found that health-related QoL was lower than that of the general population and similar to that of patients with chronic illnesses or other rare diseases, such as cystic fibrosis or hemophilia [11]. TGCT was also associated with high health care costs, mainly attributable to surgery.

When feasible, complete surgical resection of the tumor is the current standard of care for TGCT [1]. Systemic treatment with tyrosine kinase inhibitors (TKIs) that target CSF1R-mediated signaling is also used in patients for whom surgery is not an option [1]. In the TOPP registry, patients who received systemic treatment at baseline and remained on systemic treatment through 1 year of follow-up showed numerical improvements in various patient-reported outcome (PRO) measures versus those who switched to a different treatment strategy [12].

The potential impact of TGCT on function and QoL, particularly in those with D-TGCT, highlights the need for optimized therapies. This review describes the history and recent developments in the medical management of TGCT, with a focus on CSF1R inhibitors.

## History of TGCT Treatment

The treatment principles for TGCT include management within expert centers by an experienced multidisciplinary team, including pathologists, radiologists, orthopedic surgeons, oncologists, and pain specialists [1]. Shared decision making between the patient and their care team should weigh the risks and benefits of therapy and determine the best course of treatment [9].

### Surgery

Surgery is the primary treatment for TGCT, using either arthroscopic or open excision techniques [13]. Resection of the tumor with marginal excision is recommended for L-TGCT and can be curative for most patients [1, 14]. For D-TGCT, extensive synovectomy is preferred when macroscopically complete resection can be achieved without significant morbidity [1]. However, complete removal of diffuse tumors can be challenging, and many patients with TGCT experience recurrence and may require repeat surgery [2, 15, 16]. Recurrence rates following surgical resection in D-TGCT are estimated to be 20-50% [15]. Compared with complete resections, incomplete resections in patients with D-TGCT are associated with poor radiologic and clinical outcomes [16]. Furthermore, multiple surgeries can significantly impact physical functioning and QoL [10]. In some severe cases, more invasive surgeries, such as joint replacement or amputation, may be necessary [1, 2]. Tumor recurrence rates are generally low after joint replacement; however, because arthroplasties frequently need to be revised, joint replacement surgery in a young patient may result in subsequent surgery later in life [17, 18].

### Active Surveillance

Active surveillance, or a “wait and see” approach, is recommended for asymptomatic patients or in symptomatic patients if there is a risk of significant morbidity or side effects with surgery or systemic treatment [1, 19]. Findings from a retrospective study of 61 patients with treatment-naive D-TGCT suggest that active surveillance can be a safe and effective option for disease management [19]. At a median follow-up of 28 months, most patients remained stable (43%) or improved (21%) under active surveillance, whereas 37% had clinical deterioration. The frequency of follow-up during active surveillance should be individualized based on tumor location, growth pattern, and symptom severity [1].

### Radiation Therapy

Radiation therapy, including radiosynoviorthesis and external beam radiotherapy, has been used alone or as an adjuvant to surgery. Still, there are limited data on these treatments’ efficacy in TGCT [1, 13]. There are also concerns about potential safety risks, such as early-onset arthritis, joint stiffness, fibrosis, radiation-induced sarcoma, and malignant transformation [1, 13]. Therefore, radiation therapy is generally not recommended for patients with TGCT and is not used by most experts in the field [1, 14].

### Systemic Therapy

Research on the underlying molecular mechanisms of TGCT has led to the development of systemic therapies that target a specific signaling pathway [2] (Table 1). Systemic therapies are essential for the treatment of advanced or recurrent TGCT, and they may be the only option for patients with D-TCGT when surgery is not feasible due to the high risk of morbidity [1, 13]. Blockade of the CSF1/CSF1R axis using small molecule TKIs or monoclonal antibodies has proven to be an effective strategy in clinical studies of TGCT [1]. Agents that target the CSF1R are reviewed in the following section.

**Table 1:** Systemic therapies for TGCT in clinical development

Drug	MOA	Status of development
Pexidartinib	Small-molecule inhibitor of CSF1R	US FDA approved [27]
Vimseltinib	Small-molecule inhibitor of CSF1R	US FDA approved [46]
Pimicotinib	Small-molecule inhibitor of CSF1R	Phase 3 (ClinicalTrials.gov Identifier: NCT05804045)
Nilotinib	Small-molecule inhibitor of CSF1R	Phase 2 (ClinicalTrials.gov Identifier: NCT01207492)
HMPL-653	Small-molecule inhibitor of CSF1R	Phase 1 (ClinicalTrials.gov Identifier: NCT05277454)
Emactuzumab	Monoclonal antibody against CSF1R	Phase 3 (ClinicalTrials.gov Identifier: NCT05417789)
Cabiralizumab	Monoclonal antibody against CSF1R	Phase 1/2 (ClinicalTrials.gov Identifier: NCT02471716)
Lacnotuzumab	Monoclonal antibody against CSF1	Phase 2 (ClinicalTrials.gov Identifier: NCT01643850)
Zaltoprofen	NSAID activates PPARγ	Phase 2 (UMIN-CTR Identifier: UMIN000025901)

CSF1, colony-stimulating factor 1; CSF1R, colony-stimulating factor 1 receptor; MOA, mechanism of action; NSAID, nonsteroidal anti-inflammatory drug; PPARγ, proliferator‐activated receptor gamma; TGCT, tenosynovial giant cell tumor; UMIN-CTR, University Hospital Medical Information Network Clinical Trials Registry; US FDA, United States Food and Drug Administration

## Systemic CSF1R Inhibitors

**Fig. 1:**
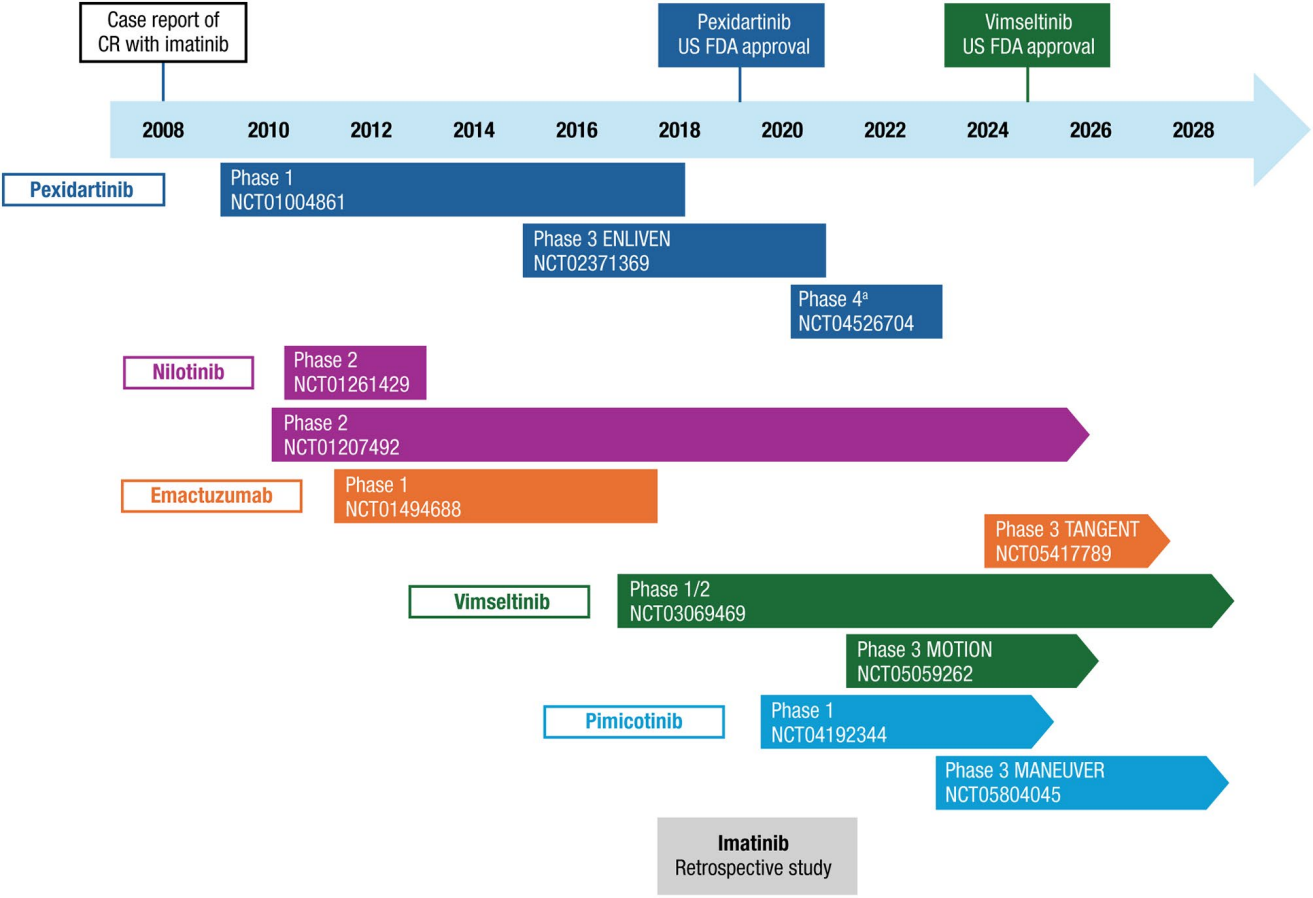
Timeline of CSF1R inhibitor studies in TGCT. CR, complete response; CSF1R, colony-stimulating factor 1 receptor; TGCT, tenosynovial giant cell tumor; US FDA, United States Food and Drug Administration. ^a^Phase 4 trial on the effects of drug discontinuation and retreatment with pexidartinib

The use of CSF1R inhibitors to treat TGCT represents a significant evolution in therapeutic strategies. Initial systemic treatments explored for TGCT were TKIs approved in oncology. The first use of a TKI with activity against CSF1R was described in a 2008 case report of a patient with relapsed TGCT who achieved complete remission with imatinib [20]. Since then, the clinical efficacy of imatinib and other CSF1R antagonists has been further investigated in TGCT (Fig. 1). A summary of key efficacy and safety data for CSF1R-targeted therapies is provided in Table 2.

**Table 2:** Summary of efficacy and safety from key CSF1R inhibitor studies in TGCT

Agent	Study design	*N*	Efficacy	Safety
Imatinib [21]	Retrospective	*N* = 58, excluding metastatic patients	ORR (RECIST): 31%	Most common AEs, any grade: • Edema/fluid retention: 48% • Fatigue: 50% • Nausea: 34% • Skin rash/dermatitis: 12% Grade 3/4 AEs: • Edema/fluid retention: 2% • Fatigue: 2% • Skin rash/dermatitis: 3% • Neutropenia: 2% • Acute hepatitis: 2% • Auditory hallucinations: 2%
Nilotinib [23, 24]	Phase 2	*N* = 56	ORR (RECIST) at 1 year: 6%	Most common (≥ 20%) AEs, grade 1/2: • Headache: 38% • Nausea: 27% • Increased ALT: 25% • Fatigue: 23% • Asthenia: 23% Grade 3 AEs: • Headache: 4% • Pruritus: 2% • Diarrhea: 2% • Increased GGT: 2% • Anorexia: 2% • Dizziness: 2% • Toxidermia: 2% • Hepatic disorder: 2%
	Phase 2 follow-up	*N* = 48	ORR (RECIST) median follow-up, 102 months: 6%	No long-term AEs reported
Emactuzumab [25, 26]	Phase 1	*N* = 28	ORR (RECIST) median follow-up, 12 months: 86%	Most common (≥ 20%) AEs, grade 1/2: • Facial edema: 64% • Asthenia: 56% • Pruritus: 56% • Rash: 40% • Peripheral edema: 36% • Nausea: 28% Grade 3 AEs: • Periorbital edema: 4% • Subacute cutaneous lupus erythematosus: 4% • Dermohypodermitis: 4% • Mucositis: 4% • Fatigue: 4%
	Phase 1 follow-up	*N* = 63	ORR (RECIST): 71% • 1-year follow-up: 70% • 2-year follow-up: 64%	Most common (≥ 30%) AEs, any grade: • Pruritus: 70% • Asthenia: 62% • Face edema: 49% • Peripheral edema: 44% • Periorbital edema: 43% • Eyelid edema: 37% • Headache: 30% Grade ≥ 3 AEs: • Pruritus: 3% • Fatigue: 3% • Periorbital edema: 2%
Pexidartinib [33, 38]	Phase 3 (ENLIVEN) • Part 1: double-blind, placebo-controlled • Part 2: open-label extension	*N* = 120 (primary analysis) *N* = 91 (final results, all pexidartinib-treated)	Primary analysis (Week 25) median follow-up, 22 months: • ORR (RECIST): 39% • ORR (TVS): 56% Final results median follow-up, 31 months: • ORR (RECIST): 60% • ORR (TVS): 68%	Most common (≥ 20%) AEs, any grade (final results): • Hair color changes: 76% • Fatigue: 47% • Nausea: 39% • Arthralgia: 37% • AST increase: 37% • Diarrhea: 32% • ALT increase: 29% • Hypertension: 29% • Dysgeusia: 28% • Rash: 28% • Peripheral edema: 26% • Periorbital edema: 24% • Headache: 23% • Pruritus 21% • Vomiting: 21% Most common (≥ 5%) grade 3/4 AEs (final results): • ALT increase: 10% • AST increase: 9% • Hypertension: 8% 3 patients experienced mixed or cholestatic hepatotoxicity in part 1
Vimseltinib [48]	Phase 3 (MOTION) • Part 1: double-blind, placebo-controlled	*N* = 123	Primary analysis (Week 25): • ORR (RECIST): 40% • ORR (TVS): 67%	Most common (≥ 20%) AEs, any grade: • Periorbital edema: 45% • Fatigue: 33% • Pruritus: 29% • Headache: 28% • Asthenia: 27% • Nausea: 25% • Blood CPK increase: 24% • AST increase: 23% Most common (≥ 5%) grade 3/4 AEs: • Blood CPK increase: 10% • Hypertension: 5%
Pimicotinib [52]	Phase 3 (MANEUVER) • Part 1: double-blind, placebo-controlled	*N* = 94	Primary analysis (Week 25): • ORR (RECIST): 54%	Specific AEs not yet reported

AE, adverse event; ALT, alanine aminotransferase; AST, aspartate aminotransferase; CPK, creatine phosphokinase; CSF1R, colony-stimulating factor 1 receptor; GGT, gamma-glutamyl transferase; ORR, overall response rate; RECIST, Response Evaluation Criteria in Solid Tumors; TGCT, tenosynovial giant cell tumor; TVS, tumor volume score

### Off-label TKIs: Imatinib and Nilotinib

A retrospective study of 58 patients with locally advanced or recurrent D-TGCT who were treated with imatinib reported an overall response rate (ORR) per Response Evaluation Criteria in Solid Tumors (RECIST) of 31% after a median follow-up of 52 months [21]. Five (11%) patients experienced grade 3/4 adverse events (AEs), including neutropenia, acute hepatitis, facial edema, skin toxicity, and fatigue. More than half (59%) discontinued imatinib within 1 year, mainly due to the patient’s or physician’s decision to stop treatment.

The TKI nilotinib was evaluated in an open-label, phase 2 trial with the goal of reducing the toxicity observed with imatinib treatment in previous case reports [20, 22]. In a trial population of 56 patients with inoperable or relapsing D-TGCT, 93% were progression free after 12 weeks of nilotinib treatment [23]. The ORR by RECIST was 6% (all partial responses) during the 1-year trial period. Grade 3–related AEs occurred in 11% of patients, including headache, pruritus, diarrhea, increased gamma-glutamyl transferase concentration, anorexia, dizziness, toxidermia, and hepatic disorder. Approximately 20% of patients discontinued nilotinib within 1 year.

In a long-term analysis of the phase 2 nilotinib trial (median follow-up of 102 months), the ORR remained at 6% and 52% of patients had disease progression [24]. More than half (58%) of the patients required subsequent treatment after stopping nilotinib. No long-term AEs were observed. A phase 2 trial of nilotinib in patients with relapsed or metastatic TGCT is ongoing (ClinicalTrials.gov Identifier: NCT01207492).

### Emactuzumab

Emactuzumab is a recombinant monoclonal antibody against CSF1R [25]. In a phase 1, proof-of-concept trial in 28 patients with locally advanced D-TGCT, the ORR by RECIST was 86%, including 2 patients with complete response, at a median follow-up of 12 months [25]. Five serious AEs were reported, including periorbital edema, lupus erythematosus (2 events), erythema, and dermohypodermitis.

The ORR was 71% in a long-term follow-up of the phase 1 trial; 70% and 64% of patients were still in response at 1 and 2 years, respectively [26]. Significant improvements were also seen in PROs, such as the EQ-5D-3L QoL. Results from the ongoing phase 3 TANGENT trial (ClinicalTrials.gov Identifier: NCT05417789) will provide further data on the efficacy and safety of emactuzumab in patients with TGCT for whom surgery is not an option.

### Pexidartinib

Pexidartinib is a TKI with strong selective activity against CSF1R; it also inhibits KIT receptor tyrosine kinase and FMS-like tyrosine kinase 3–internal tandem duplication [27]. Pexidartinib was the first systemic treatment approved for use in patients with TGCT [27]. It is approved in the United States, Taiwan, and Korea for the treatment of adults with symptomatic TGCT associated with severe morbidity or functional limitations and not amenable to improvement with surgery (or other therapy in Taiwan) [27–29]. Pexidartinib received a category 1 recommendation from the National Cancer Comprehensive Network (NCCN) for the treatment of TGCT [30].

Efficacy was first demonstrated in a phase 1 pilot trial in which treatment with pexidartinib resulted in an ORR by RECIST of 52% and a median duration of response of > 8 months [31]. The most frequent AEs were fatigue, change in hair color, nausea, dysgeusia, and periorbital edema. The phase 1 extension trial included 39 patients with advanced TGCT with a median treatment duration of 17 months [32]. The ORR was 62% by RECIST and 56% by tumor volume score (TVS; a radiographic scoring system explicitly developed for TGCT). No late-emerging safety signals were observed with longer follow-up. Pexidartinib treatment resulted in symptomatic improvement based on PRO assessments (i.e., Numeric Rating Scale [NRS] questions relevant to TGCT).

Based on the preliminary phase 1 data, pexidartinib was advanced to phase 3 development. ENLIVEN was a randomized trial that included a 24-week, double-blinded, placebo-controlled phase (part 1) followed by an open-label extension (part 2) [33]. The trial included 120 patients with advanced TGCT for whom surgery was not recommended. The ORR by RECIST at Week 25 (primary endpoint) was 39% in the pexidartinib group and 0% in the placebo group (*P* < 0.0001). The ORR by TVS at Week 25 was 56% with pexidartinib versus 0% with placebo (*P* < 0.0001). The ORRs increased to 53% by RECIST and 64% by TVS in the pexidartinib group at the data cutoff (median follow-up of 22 months).

Improvement in several secondary outcomes was also observed with pexidartinib versus placebo at Week 25, including relative range of motion of the affected joint, physical functioning assessed by the Patient-Reported Outcomes Measurement Information System–Physical Function (PROMIS-PF) scale, and worst-stiffness NRS scores [33]. The improvements in physical function and joint stiffness observed with pexidartinib were sustained after 50 weeks of treatment [34].

There was no significant difference between pexidartinib and placebo in the primary assessment of pain response at Week 25, defined as ≥ 30% decrease in weekly mean Brief Pain Inventory (BPI) worst-pain NRS score and < 30% increase in narcotic analgesic [33]. In a prespecified exploratory analysis, a modest benefit in pain relief was seen with pexidartinib versus placebo using thresholds of ≥ 50% decrease or the minimum clinically important difference (≥ 2-point decrease) in weekly worst-pain NRS score (*P* = 0.02 for both threshold comparisons) [35].

In the primary analysis of ENLIVEN, the incidence of grade 3/4 AEs was 44% with pexidartinib and 12% with placebo [33]. The most common (≥ 5%) grade 3/4 AEs with pexidartinib were increased aspartate aminotransferase (AST), alanine aminotransferase (ALT), and alkaline phosphatase (ALP) and hypertension. In the pexidartinib group, 38% of patients had dose interruptions or reductions, and 13% discontinued treatment due to AEs. Three patients treated with pexidartinib had liver enzyme elevations indicative of mixed or cholestatic hepatotoxicity (AST and ALT elevations ≥ 3 times the upper limit of normal [ULN], with bilirubin and ALP ≥ 2 times the ULN).

The long-term outcomes of pexidartinib treatment were evaluated in a pooled analysis of data from ENLIVEN and the phase 1 extension (*N* = 130; median follow-up of 39 months at the data cutoff of May 2019) [36]. The ORR was 60% by RECIST and 65% by TVS; the median time to initial response was 3.4 and 2.8 months, respectively. Overall, 68% of patients had a dose interruption or reduction due to AEs and 53% discontinued treatment, mainly due to AEs (24%). The most common AEs were similar to those reported at the original data cutoff of ENLIVEN. The majority (92%) of patients experienced aminotransferase elevations. There were 4 (3%) cases of mixed or cholestatic hepatotoxicity that all occurred within the first 8 weeks of treatment; all patients recovered within 1 to 7 months after treatment discontinuation. The hepatotoxic effects of pexidartinib were further explored in a pooled analysis of all patients in the clinical program (*N* = 140; median follow-up of 39 months) [37]. As with previous findings, most (91%) patients had dose-dependent aminotransferase elevations that resolved with treatment interruption. Most (66%) ALT or AST increases were ≥ 1 to < 3 times the ULN. Five (4%) patients had mixed or cholestatic hepatotoxicity; all recovered after discontinuing pexidartinib treatment.

Results from the final analysis of ENLIVEN provided further evidence of the long-term efficacy and safety of pexidartinib [38]. With a median follow-up of 31 months (final cutoff of April 2021), the ORR was 60% by RECIST and 68% by TVS, demonstrating that responses deepened over time since the primary analysis. The improvements in PROs were also sustained. There were no new safety signals with longer follow-up and no additional mixed or cholestatic hepatotoxicity cases.

There were 3 patients under observation in the trial who underwent surgery post-treatment per symptomatic and radiologic findings. Of note, a case report of a 32-year-old patient with D-TGCT demonstrated the successful use of upfront pexidartinib after it was determined that surgery would cause signficant morbidity and residual disease [39]. The patient achieved complete response after 2 years of treatment, with reduced pain, improved mobility, and no reported side effects.

The approval of pexidartinib was based on the results from the phase 3 ENLIVEN trial. In the United States, the prescribing information includes a boxed warning for hepatotoxicity [27]. Approval was conditional on pexidartinib only being available to patients through a Risk Evaluation and Mitigation Strategy (REMS), the TURALIO REMS (tREMS) program. A retrospective analysis of 3-year hepatic safety data from the tREMS program (*N* = 451) found that 21 (4.7%) patients met the criteria for a hepatic AE or laboratory abnormalities suggestive of a serious and potentially fatal liver injury [40]. Event onset occurred within 2 months of starting pexidartinib. Treatment was interrupted in 1 patient and 20 permanently discontinued pexidartinib. Overall, the hepatic safety data were consistent with the ENLIVEN trial results and no new safety signals were identified.

A survey of 83 patients enrolled in the tREMS program found that 29 (34.9%) required a dose reduction from the index dose and 8 (9.6%) required a dose reduction after titrating up to a higher dose [41]. The median time on pexidartinib was 6 months. At the time of the survey, 12 patients had temporarily stopped taking pexidartinib and 10 had permanently discontinued, mainly due to physician suggestion, abnormal laboratory results, or side effects. The majority (78.3% and 77.1%) of patients reported improvement in overall joint symptoms and physical function while taking pexidartinib. Significant improvements were also reported in mean worst-stiffness and worst-pain NRS scores. Results from a follow-up survey completed by 31 patients still taking pexidartinib (mean follow-up of 1 year from the baseline survey) showed sustained improvement in symptoms and treatment satisfaction; 85.7% reported improvement in overall symptoms [42].

The effects of discontinuing and then restarting pexidartinib treatment were evaluated in a phase 4 trial that included patients who had benefited from pexidartinib in a previous clinical trial [43]. Of the 32 patients enrolled, 21 chose to continue taking pexidartinib and 11 discontinued treatment with the option to restart pexidartinib. During the treatment-free period, 6 of the 11 (54.5%) patients had progressive disease by RECIST and the median progression-free survival was 22.8 months (95% confidence interval, 1.6-not estimable). Three of the 11 (27.3%) patients restarted pexidartinib and achieved disease stabilization within 6 months. There were no cases of disease progression among the 21 patients who continued pexidartinib over the 24-month trial period. The safety profile of pexidartinib was consistent with that observed in previous trials. No cases of hepatotoxicity were reported. These results suggest that pexidartinib can be safely and effectively restarted in patients who experience clinical benefit and may choose or need to discontinue treatment.

### Vimseltinib

Vimseltinib is a highly selective inhibitor of CSF1R designed with the goal of reducing potential off-target effects [44, 45]. Based on the phase 3 MOTION trial results, vimseltinib was recently approved in the United States for the treatment of adults with symptomatic TGCT for which surgical resection could potentially worsen functional limitation or cause severe morbidity [46]. The NCCN recognizes vimseltinib as a class 1 treatment option for TGCT [30].

In a first-in-human phase 1 trial of 32 patients with TGCT not amenable to surgery, vimseltinib treatment (median duration of 25.1 months) resulted in an ORR by RECIST of 72% [45]. Most nonlaboratory AEs were mild or moderate in severity. The most common grade 3/4 AEs occurring in ≥ 5% of patients were increased blood creatine phosphokinase (CPK), AST, lipase, and amylase concentrations and hypertension. There were no cases of cholestatic hepatotoxicity or drug-induced liver injury.

Preliminary data from 46 patients enrolled in an ongoing phase 2 expansion trial were consistent with the phase 1 results [47]. The median treatment duration was 15.9 months and the ORR by RECIST was 60%. Most responses were achieved ≤ 6 months after initiating vimseltinib. After 25 weeks, 48% and 52% of patients reported ≥ 30% reductions in BPI worst pain and average pain, respectively. The most common grade 3/4 AEs occurring in ≥ 5% of patients were increased CPK and hypertension. There were no signs of cholestatic hepatotoxicity. AEs led to a dose reduction or interruption in 50% and 65% of patients, respectively.

The phase 3 vimseltinib trial, MOTION, was initiated based on the phase 1/2 trial findings. Part 1 of the randomized, double-blinded, placebo-controlled MOTION trial enrolled 123 patients with TGCT not amenable to surgery [48]. The treatment period was 24 weeks, and key endpoints were assessed at the beginning of Week 25. The ORR by RECIST (primary endpoint) was 40% with vimseltinib and 0% with placebo (*P* < 0.0001). The ORR by TVS was 67% with vimseltinib versus 0% with placebo (*P* < 0.0001).

Vimseltinib was associated with significant and clinically meaningful improvement versus placebo in all key secondary outcomes, including active range of motion, PROMIS-PF, worst-stiffness NRS, EuroQoL Visual Analogue Scale, and BPI worst-pain response [48]. Functional and symptomatic improvements were observed in patients with objective responses and in those with stable disease.

The incidence of treatment-related grade 3/4 AEs was 30% in the vimseltinib group and 3% in the placebo group [48]. The most common treatment-related grade 3/4 AEs with vimseltinib were increased CPK (10%) and hypertension (5%). There was no evidence of cholestatic hepatotoxicity or drug-induced liver injury, consistent with the phase 1/2 trial. Overall, 11% of patients discontinued vimseltinib prior to Week 25. Dose reductions, interruptions, or treatment discontinuation due to AEs occurred in 42%, 53%, and 6% of patients, respectively.

As noted, vimseltinib is approved in the United States [46]. The prescribing information includes warnings for potential increases in serum creatinine without affecting renal function and hepatotoxicity [46]. A postmarket registry is required to characterize the long-term risk of hepatotoxicity [49]. Results from the open-label period (part 2) of MOTION and the long-term extension will provide additional data on the long-term efficacy and safety of vimseltinib in adults with TGCT.

### Pimicotinib

Pimicotinib is a highly selective TKI of CSF1R currently in phase 3 development for adults with TGCT [50]. Pimicotinib demonstrated efficacy in a phase 1 dose-escalation trial in patients with advanced solid tumors [51]. As of December 2022, the trial included 49 patients with TGCT. The ORR by RECIST was 77.4% with the 50 mg dose and 40.0% with the 25 mg dose, with a median treatment duration of 7.9 months. The most common AEs occurring in ≥ 20% of patients were increased lactate dehydrogenase, CPK, α-hydroxybutyrate dehydrogenase, AST, amylase, and ALT; pruritus; and rash. No serious liver injuries were reported.

The phase 3 pimicotinib trial, MANEUVER, is a randomized, double-blinded, placebo-controlled trial in adults with TGCT not amenable to surgery [50]. In part 1, patients received pimicotinib or placebo for 24 weeks. Initial topline results for the 94 patients enrolled in part 1 of the trial showed an ORR by RECIST of 54.0% for pimicotinib and 3.2% for placebo at Week 25 (*P* < 0.0001) [52]. Significant improvements in worst-stiffness NRS and BPI scores were also observed with pimicotinib treatment. The safety profile was consistent with previous reports, and there was no evidence of cholestatic hepatotoxicity. AEs led to dose reduction or discontinuation in 7.9% and 1.6% of patients, respectively. Pimicotinib will be further studied in MANEUVER’s open-label phase (part 2) and open-label extension (part 3).

## Real-world Treatment Patterns

The treatment patterns of targeted systemic therapies were recently described in a retrospective analysis of prescription and medical claims data for adults with TGCT treated between 2018 and 2021 in the United States [53]. Approximately one-quarter (23.8%) of the patients were treated with pexidartinib and 76.2% received another systemic therapy, including imatinib, sorafenib, sunitinib, and nilotinib. The probability of remaining on treatment 1 year after initiation was 54.0% for pexidartinib and ranged from 17.1 to 36.4% for the other systemic therapies. At the time of the study, pexidartinib was the only therapy approved for TGCT in the United States, indicating that many patients are treated with unapproved systemic therapies for the management of their symptoms.

## Current Recommendations

TGCT has no appreciable differences in histologic features, and it is difficult to predict the associated symptoms [54]. Even individuals with a massive disease of the knee may be relatively asymptomatic without pain and can live an active lifestyle. When this is not the case, consensus guidelines recommend surgery as the standard treatment for symptomatic TGCT [1, 14]. L-TGCT should be excised; patients who undergo L-TGCT excision generally have good outcomes [1]. For patients with D-TGCT, active surveillance may be considered if they can manage their symptoms [1]. CSF1R inhibitors are recommended if the tumor is “unresectable” or if surgery would be associated with significant morbidity. As with all therapies, the potential benefits and risks of treatment, including impact on QoL, must be carefully considered [1], taking into account patient age, lifestyle, and radiographic features. For example, some elderly patients who already have severe osteoarthritis may benefit from joint replacement surgery [1]. The hip and knee are common locations for TGCT, and hip replacement surgery is more frequently needed than knee replacement, likely due to less space around the hip joint [1] and the fact that it is frequently more symptomatic than the knee.

## Supporting the Patient Journey

Patient advocacy groups, such as TGCT Support [55], play an important role in raising disease awareness and have already helped to improve the patient journey. Access to resources and educational materials, such as plain language summaries of trial publications [56, 57], can improve the patient’s understanding of treatment options and promote engagement in health care decisions.

## Conclusions

The TGCT treatment landscape has evolved significantly, moving from a reliance on surgical intervention and symptom management to a focus on multidisciplinary team management. Targeted systemic therapies have improved tumor response, functioning, and symptoms in patients with advanced disease. The CSF1R inhibitors pexidartinib and vimseltinib are approved in the United States for adult patients with TGCT not amenable to surgery, and several other agents are in clinical development. Potential risks and benefits, including the impact on patient experience and QoL, should be carefully considered when determining the best course of treatment.

## Key References

- Wagner AJ, Tap WD, Bauer S, Blay J-Y, Desai J, Gelderblom H, et al. Long-term efficacy and safety of pexidartinib in patients with tenosynovial giant cell tumor: final results of the ENLIVEN study. The Oncologist. 2025 [in press].
  - This manuscript reports the final results of the phase 3 ENLIVEN trial of pexidartinib. Pexidartinib is FDA approved for the treatment of adults with symptomatic TGCT associated with severe morbidity or functional limitations and not amenable to improvement with surgery.
- Gelderblom H, Bhadri V, Stacchiotti S, Bauer S, Wagner AJ, van de Sande M, et al. Vimseltinib versus placebo for tenosynovial giant cell tumour (MOTION): a multicentre, randomised, double-blind, placebo-controlled, phase 3 trial. Lancet. 2024;403:2709–19. 10.1016/S0140-6736(24)00885-7.
  - This manuscript reports the primary results of the phase 3 MOTION trial of vimseltinib. Vimseltinib is FDA approved for the treatment of adults with symptomatic TGCT for which surgical resection will potentially cause worsening functional limitation or severe morbidity.
- Stacchiotti S, Durr HR, Schaefer IM, Woertler K, Haas R, Trama A, et al. Best clinical management of tenosynovial giant cell tumour (TGCT): a consensus paper from the community of experts. Cancer Treat Rev. 2023;112:102491.
  - These consensus guidelines provide evidence-based recommendations for the management of TGCT.

## Data Availability

No datasets were generated or analysed during the current study.
